# Refractory Atrial Fibrillation With Conversion Pauses in Extensive-Stage Small Cell Lung Carcinoma Presenting With Hypoxic Respiratory Failure: A Case Report

**DOI:** 10.7759/cureus.112051

**Published:** 2026-07-04

**Authors:** Ahmed R Abdelwahed, Sandesh Sharma, Madonna Phillip

**Affiliations:** 1 Internal Medicine, MountainView Regional Medical Center, Las Cruces, USA; 2 Internal Medicine, Community Health Systems - MountainView Regional Medical Center, Las Cruces, USA

**Keywords:** atrial fibrillation, case report, hypoxic respiratory failure, pleural effusion, post-obstructive pneumonia, small-cell lung carcinoma, tachy-brady syndrome

## Abstract

Small cell lung carcinoma (SCLC) is a high-grade pulmonary neuroendocrine carcinoma strongly associated with smoking and commonly presents with advanced intrathoracic disease. Atrial fibrillation (AF) is common in acutely ill patients and in patients with cancer; however, causal attribution in an individual patient is often difficult because multiple arrhythmogenic stressors coexist. A 72-year-old man with hypertension, morbid obesity, peripheral arterial disease, heavy prior tobacco exposure, and no known prior cardiac arrhythmia or structural heart disease presented with progressive dyspnea, cough, fatigue, weight loss, and hypoxemic respiratory failure. Thoracic imaging revealed bulky mediastinal and hilar lymphadenopathy, right bronchial obstruction with collapse and consolidation of much of the right lung, a large recurrent right pleural effusion, and an additional contralateral pulmonary lesion suspicious for malignancy. Integrated pleural cytology and tissue biopsy results supported the diagnosis of extensive-stage SCLC with malignant pleural involvement in this patient. The patient was treated empirically for post-obstructive pneumonia and required repeated pleural drainage procedures. During hospitalization, he developed recurrent AF with a rapid ventricular response and asymptomatic post-conversion pauses of up to six seconds, despite preserved left ventricular systolic function and no major structural heart disease on echocardiography. Rate control with beta-blockade was limited by bradycardia and hypotension; flecainide incompletely suppressed recurrent episodes; amiodarone was later initiated because rhythm instability persisted, and alternatives were limited. Transesophageal echocardiography showed no intracardiac thrombus before the planned cardioversion, but the patient spontaneously converted to sinus rhythm before shock delivery and later reverted to AF after a coughing spell. This case highlights the complexity of managing AF in advanced thoracic malignancy complicated by hypoxemia, pleural disease, and suspected post-obstructive pneumonia.

## Introduction

Small cell lung carcinoma (SCLC), which accounts for approximately 15% of lung cancers, is strongly linked to smoking exposure and is notable for its rapid growth, early dissemination, and frequent presentation with advanced-stage thoracic disease [1]. Patients commonly present with airway obstruction, pleural effusion, or respiratory failure rather than with localized disease [2].

Atrial fibrillation (AF) is the most common sustained arrhythmia encountered in hospitalized adults, and the burden of AF increases in patients with cancer due to a combination of shared risk factors, systemic inflammation, acute illness, hypoxemia, autonomic stress, and treatment-related factors [3]. Current guidance for AF management in patients with cancer generally follows standard AF principles, but real-world application is often more complex because bleeding risk, procedures, hemodynamic instability, and organ dysfunction frequently coexist [4].

Acute pulmonary disease is a relevant trigger for AF. Pneumonia, hypoxemia, adrenergic activation, and inflammatory signaling can promote atrial electrical instability in predisposed patients [5]. In addition, prolonged post-conversion pauses after AF termination, particularly when exceeding several seconds, may indicate sinus node dysfunction or tachy-brady syndrome, which can make both rate and rhythm control substantially harder to manage [6].

The present case highlights the diagnostic and therapeutic complexity of recurrent AF with conversion pauses occurring in the setting of advanced thoracic malignancy, respiratory failure, and recurrent pleural disease.

## Case presentation

A 72-year-old man with morbid obesity, hypertension, and peripheral arterial disease treated previously with bilateral femoral angioplasties and chronic outpatient therapy with apixaban and clopidogrel presented with several months of progressive dyspnea, cough, fatigue, weakness, loss of appetite, and weight loss. He had a substantial smoking history and remote extensive occupational exposure to radiation for over 10 years in his 20s-30s as a radiologist. He had no documented history of coronary artery disease, AF, or other known arrhythmias.

**Figure 1:**
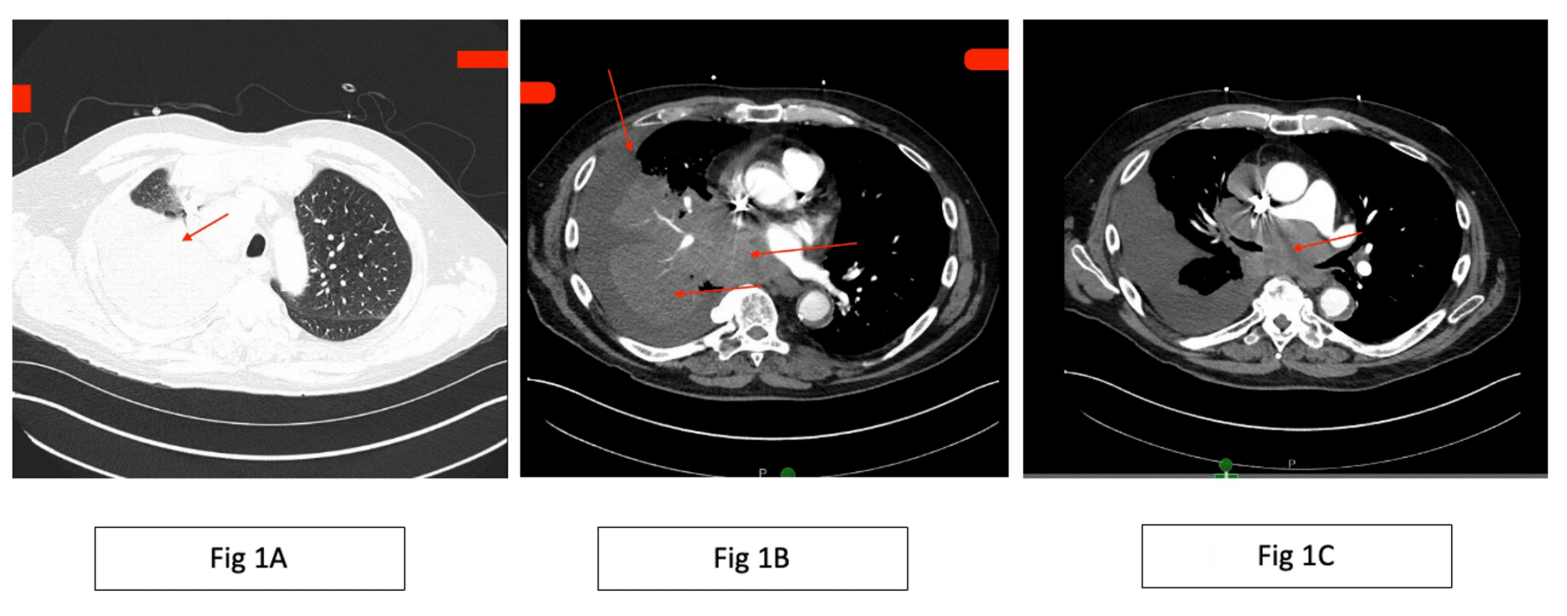
CT scan presentation on day one. Three CT scan images, including lung and soft-tissue windows, showing right-sided pleural effusion in Panel A, mediastinal/hilar adenopathy with collapsed lung and suspicious mass in Panels B and C.

Cross-sectional chest imaging revealed bulky confluent mediastinal and bilateral hilar lymphadenopathy causing right bronchial obstruction with collapse and consolidation of much of the right lung, a large right pleural effusion, and a pulmonary mass suspicious for malignancy (Figures 1A-1C).

**Figure 2:**
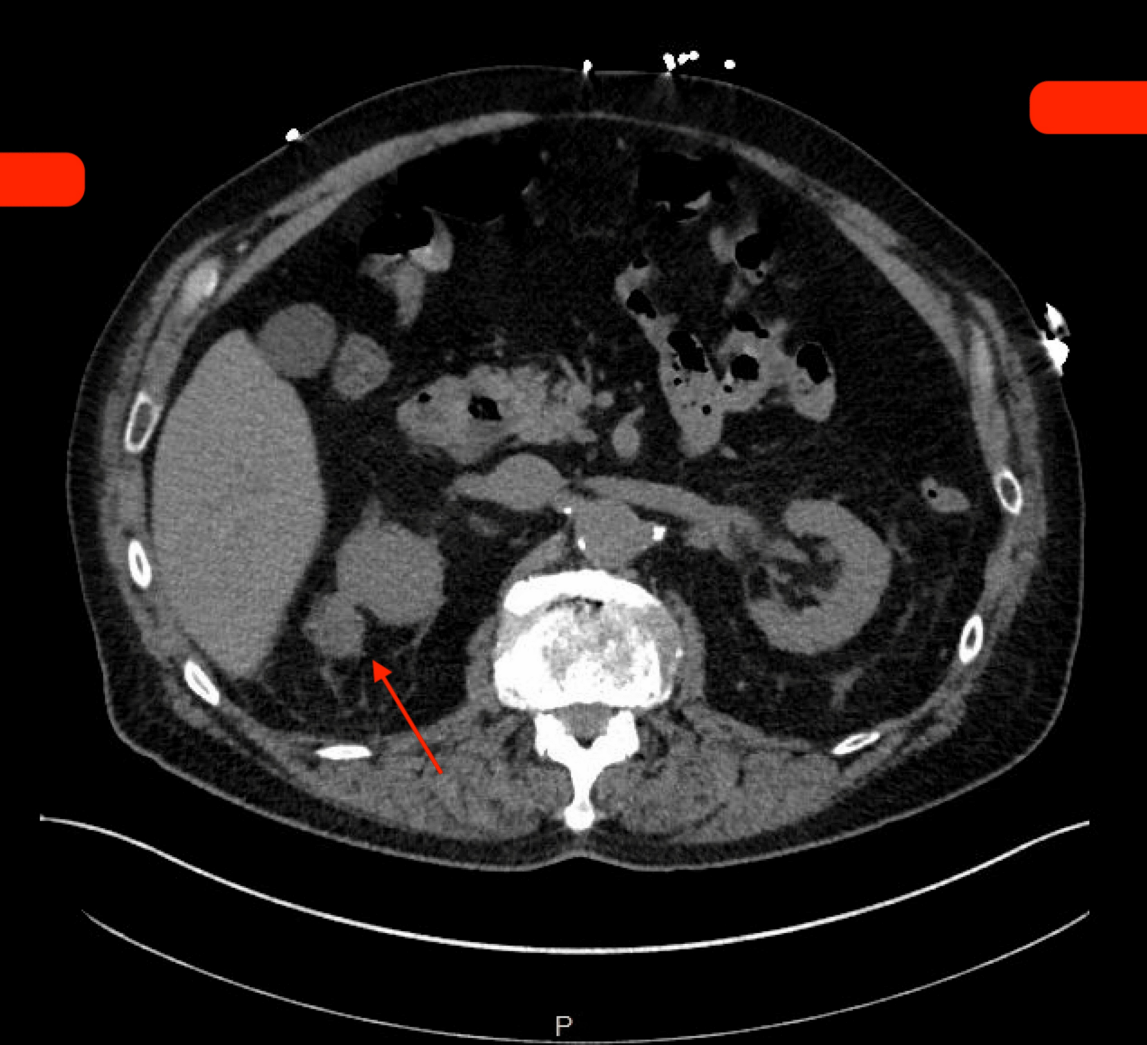
CT scan showing questionable adrenal masses that might represent metastasis.

Concurrent adrenal lesions were also observed, raising concerns regarding metastatic disease (Figure 2).

Initial laboratory testing showed mild leukocytosis (11.09 K/µL) and anemia (10.6 g/dL), while serum electrolytes, including potassium and magnesium, remained within the acceptable range during arrhythmia evaluation (Table 1).

**Table 1:** Laboratory findings on arrival. WBC = white blood cell count; LDH = lacate dehydrogenase

Laboratory test	Patient values on 12/06	Reference range
Potassium	4.2 mmol/L	3.5–5.1 mmol/L
WBC	11.09 K/µL	4.23–9.07 K/µL
Hemoglobin	10.6 mg/dL	13.0–17.0 mg/dL
Magnesium	2.07 mg/dL	1.8–2.4 mg/dL
Serum LDH	258 U/L	120–246 U/L

**Figure 3:**
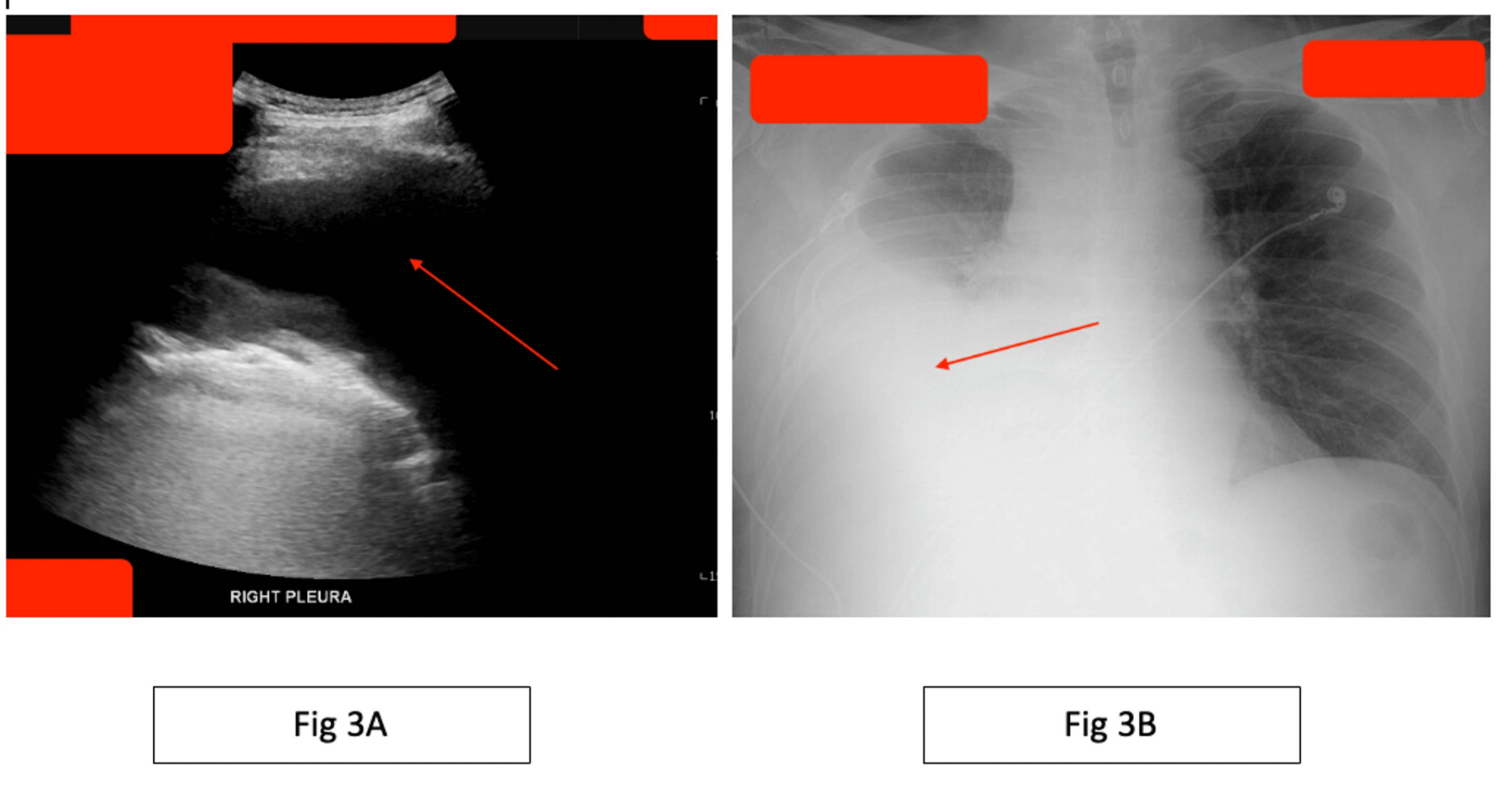
Chest ultrasound and X-ray of pleural effusion. Chest ultrasound on the right side showing pleural effusion (A). Chest X-ray showing right-sided pleural effusion (B).

Thoracentesis was performed for diagnostic clarification and symptomatic relief (Figures 3A, 3B). Pleural fluid analysis was also obtained and showed findings consistent with exudative effusion based on Light’s criteria (Table 2).

**Table 2:** Pleural fluid analysis and normal ranges for the two thoracenteses performed for the patient. WBC = white blood cell count; RBC = red blood cell count; LDH = lacate dehydrogenase

	Patient’s pleural fluid analysis on 12/16	Patient’s pleural fluid analysis on 12/29	Normal range
Pleural fluid clarity	Cloudy	Slightly Cloudy	Clear
Pleural fluid color	Yellow	Yellow	Colorless
Pleural fluid WBC	1,932 cells/mm³	1,420 cells/mm³	0–10 cells/mm³
Pleural fluid RBC	4,000 cells/mm³	5,000 cells/mm³	0–0 cells/mm³
Pleural fluid lymph	99%	92%	0-75%
Pleural fluid glucose	109 mg/dL	100 mg/dl	NA
Pleural fluid protein	3.5 g/dL	2.9 g/dl	NA
Serum protein	6.6 g/dL	5.3 g/dl	6.4-8.2 g/dl
Pleural fluid LDH	283 U/L	214 U/L	NA
Pleural fluid albumin	2.3 g/dL	1.9 g/dl	NA
Serum albumin	4.1 g/dL	3.6 g/dl	3.2-4.8 g/dl
Serum LDH	258 U/L	230 U/L	120-246 U/L

**Figure 4:**
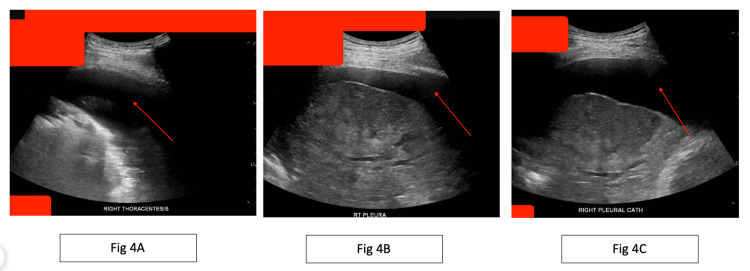
Multiple chest ultrasounds during the patient’s hospital stay. Pleural effusion can be seen reaccumulating, requiring multiple thoracenteses, all on the right side (A-C).

Pleural fluid later reaccumulated rapidly after three days, then after three more days, with a total of three thoracenteses, requiring repeated drainage and ultimately catheter-based management, a pattern consistent with persistent malignant pleural disease and sustained thoracic stress (Figures 4A-4C). Due to leukocytosis and radiographic concerns for possible post-obstructive infection, empirical levofloxacin was initiated for presumed post-obstructive pneumonia.

Bronchoscopy with tissue sampling was also performed from the right middle lobe. Bronchial wash cytology was non-diagnostic, which is not unusual given the limited sensitivity of washings alone; however, integrated pathologic assessment combining pleural fluid cytology and biopsy findings supported an extensive-stage SCLC with malignant pleural involvement. Pathology report revealed degenerated endobronchial mucosa with aggregates of crushed markedly hyperchromatic round cells that were negative for squamous marker p40, negative for Ki-67, and negative for Napsin A. Multi-cytokeratin and CK AE1/AE3 were patchily positive in the areas of atypia. These dark, round cells were negative for leukocyte common antigen. Minimal focal TTF-1 expression was identified, and the tumor stained for chromogranin with very weak focal reactivity for synaptophysin.

**Figure 5:**
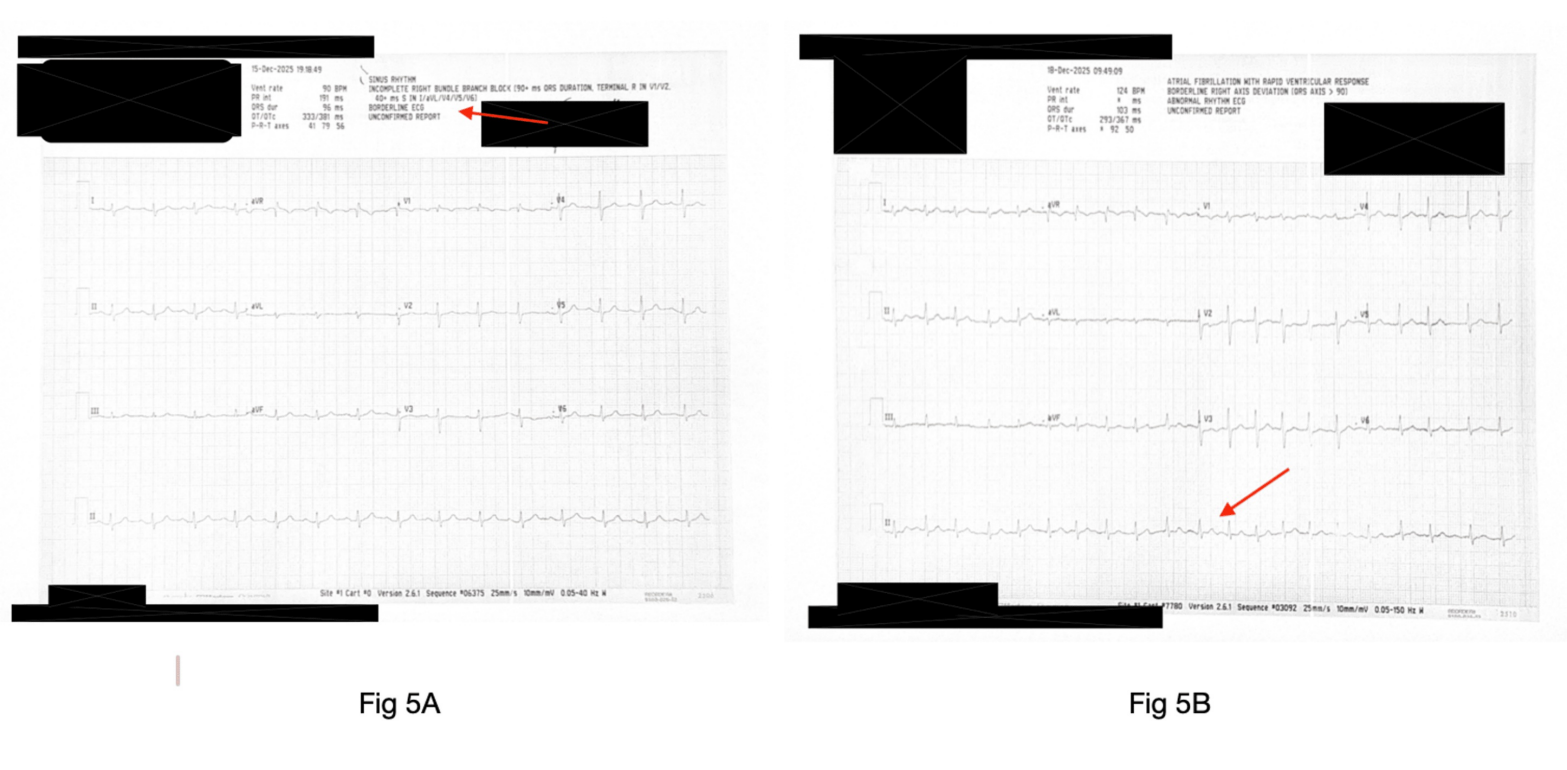
EKGs showing sinus rhythm and atrial fibrillation. EKG showing normal sinus rhythm on arrival to the hospital (A). EKG showing new-onset atrial fibrillation with rapid ventricular response (B).

During hospitalization, the patient developed recurrent paroxysmal AF, sometimes with a rapid ventricular response (Figures 5A, 5B).

**Figure 6:**
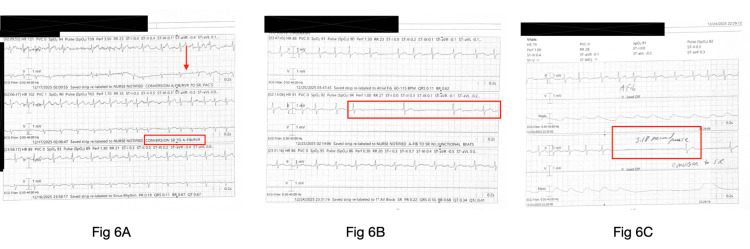
Multiple telemetry strips during the patient’s stay with different findings. Conversion from sinus rhythm to atrial fibrillation (A). Conversion from atrial fibrillation to sinus rhythm with junctional beats (B). Image showing the sinus pause (C).

Telemetry documented repeated transitions between sinus rhythm and AF, including asymptomatic post-conversion pauses, initially around three seconds (Figures 6A-6C). The longest pauses were reported during conversion from AF back to sinus rhythm, a pattern that raised concerns for tachy-brady physiology rather than isolated medication effect alone.

Transthoracic echocardiography revealed preserved left ventricular systolic function with an estimated ejection fraction of approximately 55% and no major structural abnormalities, normal left atrial size with a volume index of 18.9 mL/m². This finding did not eliminate the possibility of age-related atrial substrate or sinus node dysfunction; however, it made severe structural cardiomyopathy a less convincing explanation for rhythm instability.

Rate control was initially attempted using metoprolol; however, this strategy was limited by bradycardia, pauses, low blood pressure, and orthostatic intolerance. Flecainide was then introduced and escalated, resulting in transient improvement but no durable suppression of recurrent AF. Flecainide was considered only after the echocardiogram was reviewed and confirmed with a normal ejection fraction, no previous structural heart disease, and no coronary artery disease, especially after taking the patient’s age into consideration. Given the patient’s newly diagnosed extensive-stage SCLC and significant underlying pulmonary disease, the cardiology team initially deferred amiodarone because of concern for its potential pulmonary toxicity. However, after recurrent AF persisted despite metoprolol and flecainide, the anticipated benefits of rhythm control were felt to outweigh the potential pulmonary risks. As the arrhythmia continued to recur, amiodarone was initiated. Anticoagulation management was complicated by the need for invasive procedures, such as biopsy and pleural interventions. Although transesophageal echocardiography showed no intracardiac thrombus before the planned direct-current cardioversion, the procedure was not performed. This was because the patient spontaneously returned to sinus rhythm before the shock and reverted to AF shortly afterward during a coughing spell.

The patient remained oxygen-dependent throughout the admission period. After a multidisciplinary discussion, the patient declined cancer-directed systemic therapy and chose palliative-focused management after an extensive discussion with our oncologist. At the time of discharge, the patient continued to have AF with intermittent episodes of rapid ventricular response, with spontaneous conversion to either normal sinus rhythm or atrial fibrillation with a controlled ventricular response. He was discharged on amiodarone and metoprolol succinate daily, with holding parameters for hypotension and bradycardia. Given his advanced malignancy and decision to pursue palliative-focused care, amiodarone was intended as a pragmatic strategy for symptom control, with outpatient cardiology follow-up for reassessment of long-term rhythm management.

## Discussion

This case should not be interpreted as proof that SCLC uniquely causes AF. A more practical interpretation is that advanced thoracic malignancy creates conditions for refractory AF by combining several powerful arrhythmogenic stressors in the same patient [4].

The most convincing precipitant was acute pulmonary and thoracic illness. The patient had hypoxemic respiratory failure, extensive endobronchial obstruction, collapse and consolidation of much of the right lung, recurrent large pleural effusion, and probable post-obstructive pneumonia. Together, these conditions likely increased autonomic stress, inflammatory signaling, atrial stretch, and overall electrical instability, thereby promoting recurrent AF [4,5,7].

Within this unstable cardiopulmonary setting, post-conversion pauses add an important layer of complexity. Pauses of up to six seconds after termination of AF suggest concomitant sinus node dysfunction or tachy-brady physiology, particularly in older patients, and help explain why this was not simply a routine case of AF with a rapid ventricular response. This interpretation is more informative because it clarifies why otherwise reasonable therapies, such as beta-blockade, became difficult to use and why rhythm disturbance was assumed to be of disproportionate importance during admission. Similarly, the incidence and risk of conversion pauses in patients with AF are likely influenced by a complex interplay of systemic inflammation, comorbid cardiovascular risk factors (hypertension, diabetes, and obesity), and atrial structural remodeling, such as fibrosis. Although direct data on conversion pause incidence in patients with cancer are sparse, these interconnected factors can increase susceptibility to pauses and conduction abnormalities during rhythm conversion [8]. Focused clinical research is needed to quantify and characterize conversion pauses, specifically in cancer populations undergoing AF treatment, and to develop tailored prevention and management strategies.

Cancer itself remains relevant, but probably as a contextual amplifier rather than an isolated electrophysiologic cause, as discussed earlier. Cancer-associated AF is understood to arise from overlapping risk factors, systemic inflammation, thrombogenicity, direct organ stress, and treatment exposure [9,10]. In this patient, the advanced intrathoracic tumor burden likely intensified the respiratory and inflammatory substrates for AF, even though a direct tumor-specific mechanism could not be proven.

The neuroendocrine phenotype of SCLC should be carefully considered as it represents a theoretical contributor to arrhythmogenesis, although a tumor-specific mechanistic role could not be established in this patient. A previous study reported that 15% of patients with neuroendocrine tumors had atrial fibrillation/flutter [11]. The mechanism by which lung cancer, including SCLC, can trigger AF has been explained as secondary to inflammation, cytokines, and NLRP3 inflammasome activation in lung cancer, promoting atrial remodeling and AF susceptibility, tumor mass effect compressing the left atrium/pulmonary veins/artery triggering refractory AF with rapid ventricular response, which improved after chemotherapy and tumor shrinkage, as well as hormone and vasoactive substances secreted by the tumor [4,11,12]. The neuroendocrine phenotype of SCLC may contribute to both AF susceptibility and management complexity; however, when combined with cancer-related inflammation, thoracic disease burden, and other coexisting risk factors, the overall arrhythmogenic burden is likely to be substantially amplified. Further research is necessary to determine whether the neuroendocrine characteristics of SCLC independently increase the risk of AF and to clarify the nature of any causal relationship.

The management course also adds value to this case study. Metoprolol provided only partial control and aggravated bradycardic liability, flecainide offered incomplete suppression, and amiodarone was ultimately chosen because alternatives were limited and rhythm instability remained clinically significant, despite awareness of its pulmonary toxicity profile. This decision was clinically understandable because short-term rhythm stabilization may outweigh the longer-term pulmonary risk when alternatives fail, and the patient remains symptomatic or hemodynamically vulnerable. Therefore, this case contributes most meaningfully to the intersection of pulmonary medicine, cardiology, and oncology. This shows that recurrent AF in advanced lung cancer may be both multifactorial and management-limiting.

The main limitation of this study is that causality cannot be established. Additional limitations include the inability to exclude preexisting occult atrial disease, the absence of electrophysiological evaluation, and the presence of several plausible confounders simultaneously, including age, vascular comorbidity, infection, hypoxemia, recurrent pleural effusion, and medication. Nevertheless, the temporal relationship between severe thoracic disease and recurrent hard-to-control AF remains clinically relevant and is worthy of reporting.

## Conclusions

This case is best characterized as refractory AF with post-conversion pauses in the context of extensive-stage SCLC, hypoxemic respiratory failure, recurrent malignant pleural disease, and probable post-obstructive pneumonia. The most plausible explanation is a multifactorial arrhythmogenic process, wherein advanced thoracic malignancies exacerbate pulmonary, inflammatory, and autonomic stress. The presence of post-conversion pauses, raising concerns of tachy-brady syndrome, further limits the tolerance to conventional therapies and complicates the management. Although neuroendocrine mechanisms have been hypothesized to contribute to arrhythmogenesis in selected cancers, a direct tumor-specific mechanism could not be established in this case. Clinically, this case underscores the importance of evaluating AF in advanced thoracic malignancies through a comprehensive framework that encompasses tumor burden, hypoxemia, infection, pleural disease, intrinsic conduction system vulnerability, and potential neuroendocrine tumor-related hormonal effects.

## References

[1] Zhang Y, Vaccarella S, Morgan E (2023). Global variations in lung cancer incidence by histological subtype in 2020: a population-based study. Lancet Oncol.

[2] Kim SY, Park HS, Chiang AC (2025). Small cell lung cancer: a review. JAMA.

[3] Leiva O, AbdelHameid D, Connors JM, Cannon CP, Bhatt DL (2021). Common pathophysiology in cancer, atrial fibrillation, atherosclerosis, and thrombosis: JACC: CardioOncology state-of-the-art review. JACC CardioOncol.

[4] Davis NE, Prasitlumkum N, Tan NY (2024). Atrial fibrillation and cancer-epidemiology, mechanisms, and management. J Clin Med.

[5] Maisano A, Vitolo M, Imberti JF (2022). Atrial fibrillation in the setting of acute pneumonia: not a secondary arrhythmia. Rev Cardiovasc Med.

[6] Su L, Wang X, Kang F, Gong C, Chen D (2024). Atrial fibrillation ablation compared to pacemaker therapy in patients with tachycardia-bradycardia syndrome: a systematic review and updated meta-analysis. Medicine (Baltimore).

[7] Leventopoulos G, Koros R, Travlos C (2023). Mechanisms of atrial fibrillation: how our knowledge affects clinical practice. Life (Basel).

[8] Fabiani I, Colombo A, Bacchiani G, Cipolla CM, Cardinale DM (2019). Incidence, management, prevention and outcome of post-operative atrial fibrillation in thoracic surgical oncology. J Clin Med.

[9] Awashra A, Abdul-Hafez HA, Swalha M (2025). Cancer, inflammation, and thrombosis: drivers of atrial fibrillation in oncology patients. Clin Appl Thromb Hemost.

[10] Hajjar LA, Fonseca SM, Machado TI (2021). Atrial fibrillation and cancer. Front Cardiovasc Med.

[11] Doshi K, Iyer N, Kasire SP (2025). Hospital outcomes in patients with neuroendocrine tumors and atrial-fibrillation/flutter. J Clin Oncol.

[12] Emmanuel KE, Jensen N, Anyanwagu U (2021). Refractory atrial fibrillation with rapid ventricular rate in a patient with small cell carcinoma of the lung encasing the right pulmonary artery: a case report and insight into therapeutic options. Cureus.

